# Photoresponsive peptide materials: Spatiotemporal control of self-assembly and biological functions

**DOI:** 10.1063/5.0179171

**Published:** 2023-12-18

**Authors:** Kazunori Matsuura, Hiroshi Inaba

**Affiliations:** 1 Department of Chemistry and Biotechnology, Graduate School of Engineering, Tottori University, Koyama-Minami 4-101, Tottori 680-8552, Japan; 2 Center for Research on Green Sustainable Chemistry, Tottori University, Koyama-Minami 4-101, Tottori 680-8552, Japan

## Abstract

Peptides work as both functional molecules to modulate various biological phenomena and self-assembling artificial materials. The introduction of photoresponsive units to peptides allows the spatiotemporal remote control of their structure and function upon light irradiation. This article overviews the photoresponsive peptide design, interaction with biomolecules, and applications in self-assembling materials over the last 30 years. Peptides modified with photochromic (photoisomerizable) molecules, such as azobenzene and spiropyran, reversibly photo-controlled the binding to biomolecules and nanostructure formation through self-assembly. Photocleavable molecular units irreversibly control the functions of peptides through cleavage of the main chain and deprotection by light. Photocrosslinking between peptides or between peptides and other biomolecules enhances the structural stability of peptide assemblies and complexes. These photoresponsive peptides spatiotemporally controlled the formation and dissociation of peptide assemblies, gene expressions, protein–drug interactions, protein–protein interactions, liposome deformation and motility, cytoskeleton structure and stability, and cell functions by appropriate light irradiation. These molecular systems can be applied to photo-control biological functions, molecular robots, artificial cells, and next-generation smart drug delivery materials.

## I. INTRODUCTION

Peptides, which are short protein fragments, not only act as functional molecules that modulate many biological phenomena, such as protein–protein interaction,^1–3^ gene expression,^4–6^ cytoskeleton dynamics,^7^ and amyloid fibril formation,^8^ but also attract attention as components for biomaterials,^9–11^ therapeutic drugs,^9–14^ bioimaging probes,^15–17^ and nanomaterials.^18–20^ Biomaterials composed of peptides can be mainly used for tissue engineering scaffolds, drug delivery vehicles, and three-dimensional printing bioinks.^9–11^ Cell-penetrating peptides and organelle-localized peptides play a crucial role in efficient drug delivery systems into cells and bioimaging in cells.^21,22^ Peptides that bind to metals and semiconductor materials serve as an interface for adhesion between inorganic and biological materials.^23^ Rationally designed peptides that self-assemble through the formation of secondary structures, such as α-helices and β-sheets, can be used to models of amyloid fibril formation, gel materials for tissue engineering, self-healing materials, nanocapsules, and nanotubes for drug delivery.^23–30^ These functional peptide materials can be synthesized using solid-phase peptide synthesis and ligation technology between peptides, based on the rational design.^31,32^ Selecting peptides with desired functions from biosynthesized random libraries is possible using phage display and other peptide display technologies.^33^

**FIG. 1.**
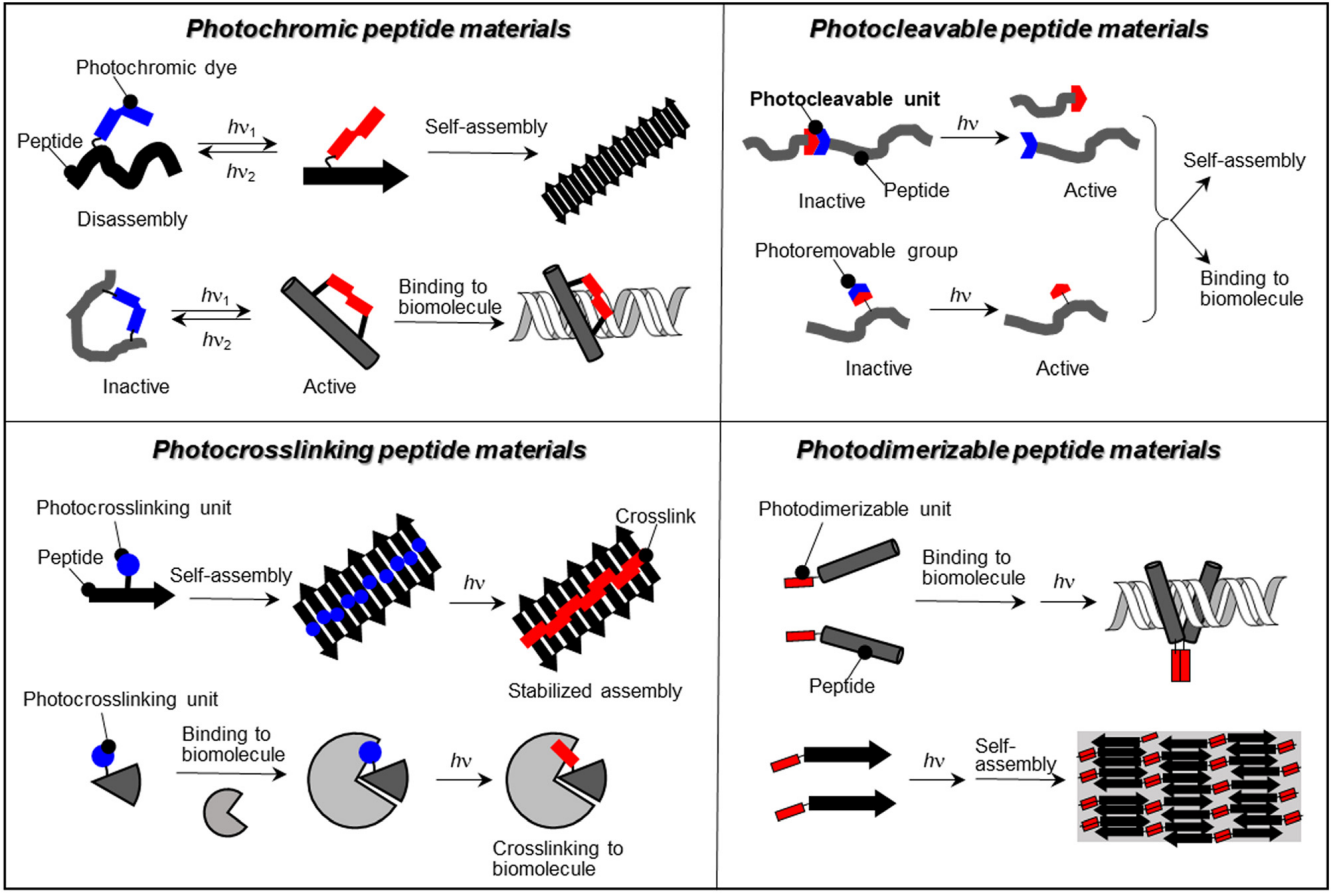
Overview of photoresponsive peptide materials.

**FIG. 2.**
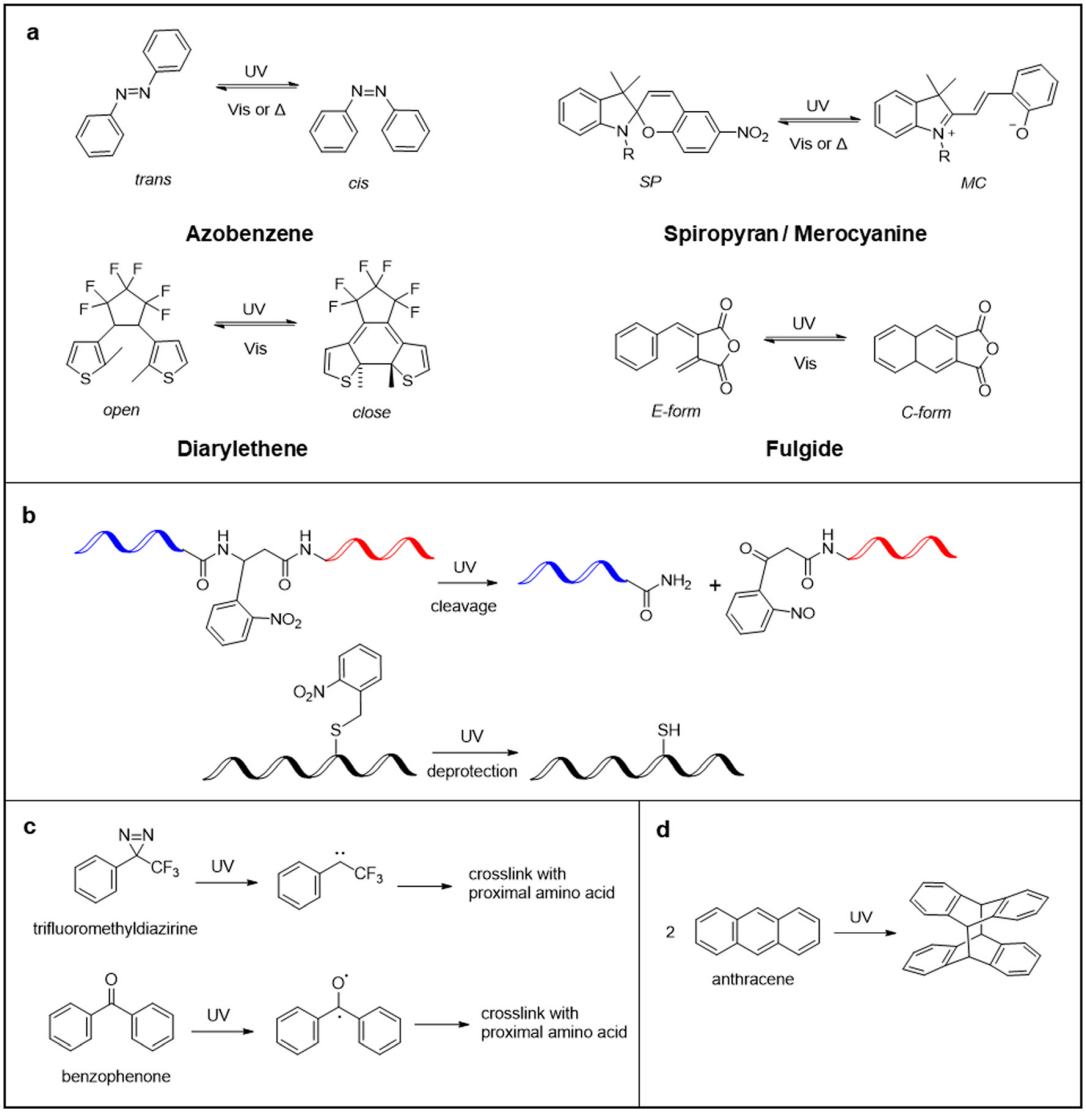
Examples of molecular units for photoresponsive peptide materials. (a) Photochromic, (b) photocleavable, (c) photocrosslinking, and (d) photodimerizable molecular units.

Imparting stimulus-responsiveness, such as pH, temperature, light, metal ions, and enzymes, to peptides and proteins allows them to be equipped with smart features to control biological activity, drug release, and nanomaterial properties.^11^ In particular, the introduction of photoresponsive units to peptides and proteins allows the spatiotemporal remote control of their structure and function upon light irradiation.^34–38^ Light is a clean energy source with low biological toxicity that can be endowed with a range of capabilities due to its lack of waste formation, wavelength tunability, recyclability, and potential for spatiotemporal resolution. Photostimulation with such advantageous properties enables even photocontrol of the structure and function of peptides and proteins in living systems.^39,40^ Photochromic (photoisomerizable), photocleavable, photocrosslinking, and photodimerizable peptide materials are the most prominent design strategies for photoresponsive peptide materials (Fig. 1). This review presents an overview of each photoresponsive peptide material, especially photocontrol of peptides, regarding self-assembly, biomolecule interaction, such as proteins and nucleic acids, and cytoskeleton functions. Figure 2 summarizes the available photoresponsive molecular tools.^41–46^ Azobenzene, which is most prominently used as a photoswitch, isomerizes from the *trans* state to the *cis* state by ultraviolet (UV) light irradiation, and returns to the *trans* state by visible light irradiation or heat [Fig. 2(a)].^43,44^ Similarly, spiropyran isomerizes to merocyanine by UV light irradiation and reverts to spiropyran by visible light irradiation or heat.^45,46^ The isomerization between uncharged spiropyran and zwitterionic merocyanine induces large structural changes in the peptides. Photochromic diarylethenes and fulgides are advantageous by being able to isomerize only with light of different wavelengths and not thermally.^41,42,45^ Photocleavable molecular units, which are typified by *o*-nitrobenzyl groups, irreversibly control the functions of peptides by main chain cleavage and light deprotection.^41,47^ Photocrosslinking of peptides or peptides and other biomolecules increases the structural stability of peptide assemblies and complexes and allows the profiling of peptide-biomolecular interactions.^48^

## II. PHOTOCHROMIC PEPTIDE MATERIALS

### A. Peptide assemblies controlled by photoisomerization

**FIG. 3.**
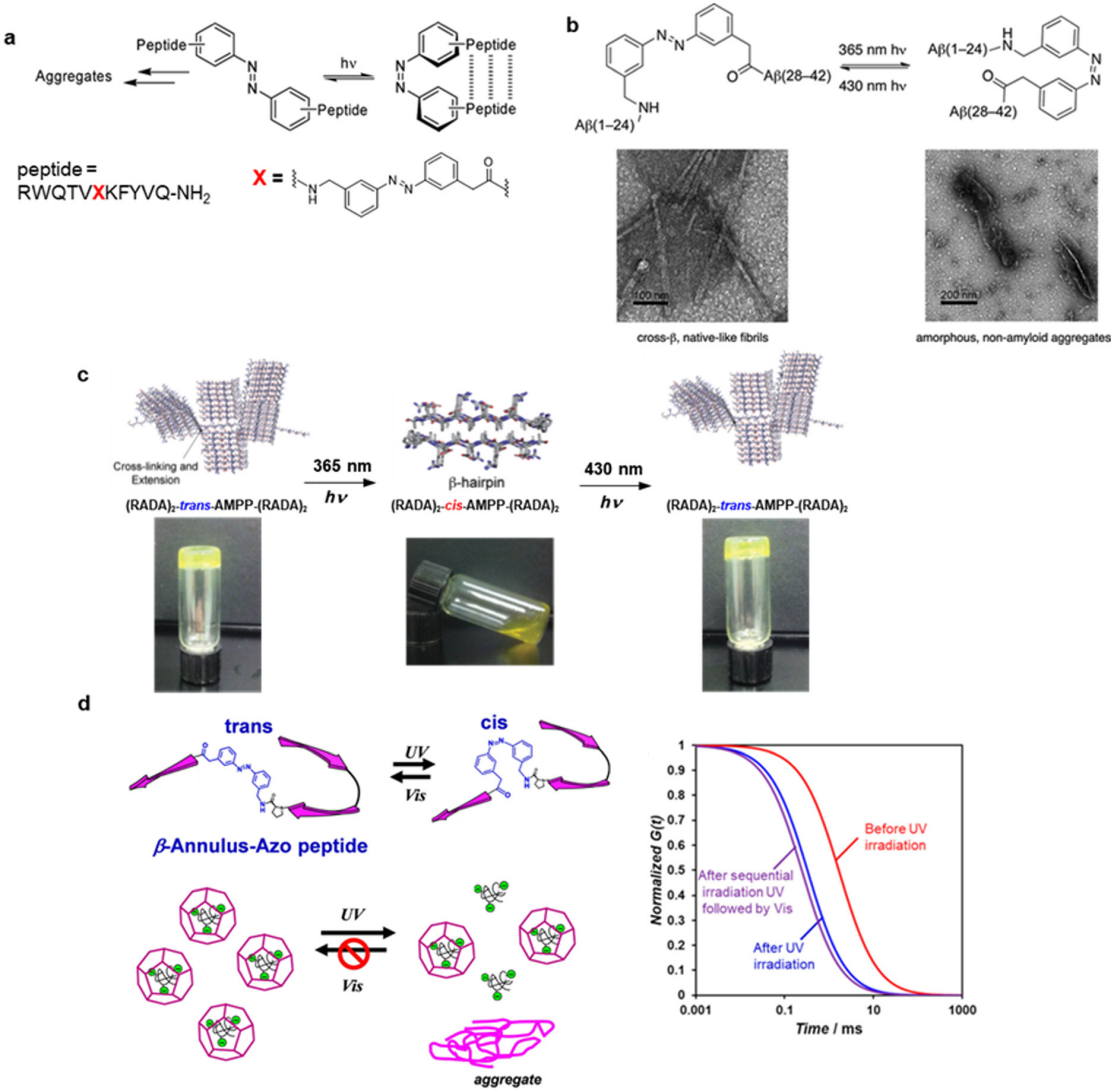
Photoisomerization of azobenzene controls peptide assemblies. (a) β-hairpin structure controlled by azobenzene-containing peptide photoisomerization. Reproduced with permission from Aemissegger *et al.*, J. Am. Chem. Soc. **127**, 2929–2936 (2005). Copyright 2005 American Chemical Society. (b) Cross-β amyloid fibril formation by photoisomerization of azobenzene-containing Amyloid-β mimic peptide. Reproduced with permission from Doran *et al.*, ACS Chem. Neurosci. **3**, 211 − 220 (2012). Copyright 2012 American Chemical Society. (c) Photocontrol of hydrogel viscoelasticity comprising of azobenzene-containing (RADA)_4_ peptide. Reproduced with permission from Doran *et al.*, Polym. Chem. **5**, 241–248 (2014). Copyright 2014 Royal Society of Chemistry. (d) Guest-release photocontrol from artificial viral capsid self-assembled from an azobenzene-containing β-annulus peptide. FCS showed the release of fluorescent-labeled dextrans from artificial viral capsid by UV irradiation. Reproduced with permission from Matsuura and Fujita, Int. J. Mol. Sci. **22**, 4028 (2021). Copyright 2021 Authors, licensed under a Creative Commons Attribution (CC BY) License.

Fibrous peptide assemblies are useful not only for elucidating the formation mechanism of amyloid fibrils, which are involved in protein misfolding diseases, such as Alzheimer's and prion diseases, but also as self-assembling nanomaterials that can be used for self-healing materials, artificial extracellular matrices, drug delivery, and nanoelectronics. By introducing photochromic units, peptide nanofibers acquire the ability to switch their structure and function with light. Aemissegger and co-workers developed a *β*-hairpin peptide that incorporated a photochromic azobenzene linker as a turn element [Fig. 3(a)].^49,50^ The ^D^Pro-Gly segment of 12-residue peptide, derived from protein GB1, was replaced by an azobenzene linker, 3-[(3-aminomethyl)-phenylazo]phenylacetic acid (AMPP). The thermodynamically favored *trans*-form of azobenzene-containing peptide self-assembled into oligomer, whereas the photoisomerization to *cis*-form induced well-defined β-hairpin monomer formation. The turn mimic that is based on azobenzene photoswitch can serve as a photoregulatory element to control β-hairpin formation in peptide hormones or larger proteins. Waldauer *et al.* determined hexapeptide (Ac-CHGGCK-NH_2_) crosslinked with azobenzene at the two Cys residues by molecular dynamics simulations.^51^ The peptide that crosslinked with azobenzene enables reversible photo-triggered aggregation to form amyloid-like fibril in the *trans* state, whereas soluble monomer in the *cis* state. Doran *et al.* incorporated an AMPP into the putative turn region of the amyloid β 42 (Aβ42) chain as a photoswitch linker [Fig. 3(b)].^52^ The *trans* state of the azobenzene-containing Aβ42 has formed fibrous assemblies with cytotoxicity that is similar to wild-type Aβ42. In contrast, photoisomerization to the *cis* state afforded amorphous aggregate that exhibits no cytotoxicity. β-Turn nucleation has been proposed as a rate-determining step in the self-assembly pathway of Aβ fibril, but these results demonstrated insight into Aβ self-assembly that β-turn intermediates are not strictly required for Aβ fibril or cytotoxic oligomer formation.

Supramolecular hydrogels that are formed by peptide self-assembly are attractive materials for controlled drug release and regenerative medicine. Photoresponsive hydrogels have been developed by incorporating azobenzene photoswitches into the side chains or terminals of short self-assembling peptides.^53–55^ Doran *et al.* achieved reversible optical control of hydrogel viscoelasticity by incorporating the azobenzene turn mimetic (AMPP) into the center of a hydrogel-forming peptide (RADA)_4_ [Fig. 3(c)].^56^ The *trans* state formed a rigid self-supporting gel via an extended or bent β-sheet structure, while photoisomerization to the *cis* state reduced hydrogel rigidity, which was caused by disrupting the well-ordered assembly structure. Such photo-responding hydrogel of self-assembled peptides can be applied to peptide materials that allow cultured cell harvesting and encapsulated drug release by light irradiation.

A 24-residue β-annulus peptide (INHVGGTGGAIMAPVAVTRQLVGS), derived from tomato bushy stunt virus, self-assembled into hollow nanocapsules with sizes of 30–50 nm in water.^57^ A photochromic azobenzene linker, AMPP, replaced the putative turn region (Pro14-Val15-Ala16 sequence) of β-annulus peptide, which plays a pivotal role in artificial viral capsid formation [Fig. 3(d)].^58^ The *trans* state of β-annulus-azo peptide self-assembled into capsids with diameters of 30–50 nm at 25 *μ*M close to the critical aggregation concentration, whereas the *cis*-rich state after UV irradiation formed micrometer-sized aggregates. Circular dichroism (CD) spectra revealed increased contents of the random coil by photoisomerization from the *trans* to the *cis*-form. Fluorescence correlation spectroscopy (FCS) showed that fluorescein isothiocyanate (FITC)-labeled dextran was encapsulated in the artificial viral capsid that comprises *trans*-rich β-annulus-azo peptide. The FCS curve shifted toward shorter diffusion times after UV irradiation, indicating the release of FITC-labeled dextran from the capsid. However, FITC-labeled dextran re-encapsulation in the capsid was not observed after sequential UV and visible-light irradiations, due to aggregate formation of the *cis*-rich β-annulus-azo peptide. This proof-of-concept provides a guideline to develop a novel photoinduced drug delivery system using the azobenzene-containing artificial viral capsids.

**FIG. 4.**
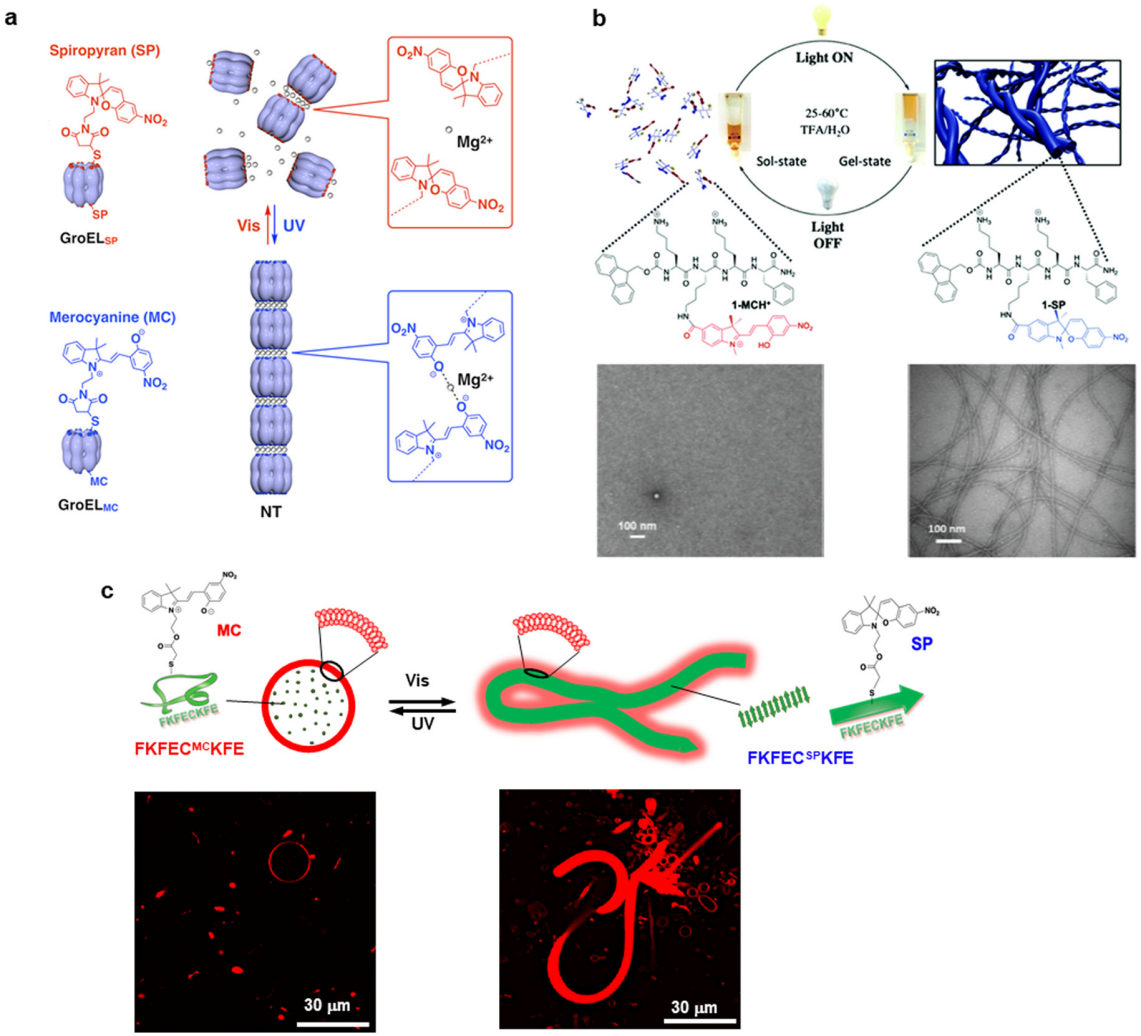
Protein/peptide assemblies controlled by spiropyran/merocyanine photoisomerization. (a) Spiropyran/merocyanine-modified GroEL photoisomerization controls protein nanotube. Reproduced with permission from Sendai *et al.*, J. Am. Chem. Soc. **135**, 11509–11512 (2013). Copyright 2013 American Chemical Society. (b) Light-driven hydrogel self-assembled from spiropyran/merocyanine-modified peptide. Reproduced with permission from Liu *et al.*, Chem. Commun. **57**, 13776–13779 (2021). Copyright 2021 Royal Society of Chemistry. (c) Peptide nanofibers controlled by photoisomerization of spiropyran/merocyanine induced dramatic morphological changes in giant liposomes. Reproduced with permission from Liang *et al.*, Front. Mol. Biosci. **10**, 1137885 (2023). Copyright 2023 Authors, licensed under a Creative Commons Attribution (CC BY) License.

Photoisomerization of uncharged spiropyran and zwitterionic merocyanine is a photoswitch system that causes more drastic structural changes than azobenzene.^46^ Sendai *et al.* developed a photoreconfigurable supramolecular nanotube comprising spiropyran/merocyanine-modified GroEL, which is one of the molecular chaperones [Fig. 4(a)].^59^ GroEL mutant containing 14 Cys residues at each cavity entrance was modified with spiropyran/merocyanine-maleimide. The merocyanine-modified GroEL was self-assembled into micrometer-long nanotubes by the coordination of Mg^2+^ with merocyanine. The nanotubes dissociated to monomeric GroEL when the merocyanine-modified GroEL was photoisomerized into the spiropyran form by visible light irradiation. Conversely, the nanotubes were reconstructed when the spiropyran-modified GroEL was exposed to UV light to convert to merocyanine form. The GroEL cavity can encapsulate macromolecules or nanoparticles, thus the spiropyran/merocyanine-modified GroEL nanotube allows the development of a spatiotemporal control system of guest delivery. Liu *et al.* demonstrated that spiropyran-modified tetrapeptide Fmoc-KK^SP^KF-NH_2_ self-assembled into β-sheet nanofibers to form a hydrogel in acidic water under visible light exposure, while dissociated when the light source was removed [Fig. 4(b)].^60^ The spiropyran-modified peptide thermally isomerized into a more stable protonated merocyanine form and the nanofiber was dissociated due to increased electrostatic repulsion in the absence of continuous visible light irradiation. This light-driven dissipative self-assembly of peptides enables simple spatiotemporal control of hydrogel materials without fuel waste production.

Cytoskeletons in eukaryotic cells, such as microtubules and actin filaments, dynamically control cellular morphology by reversible constituent protein polymerization/depolymerization. We developed an artificial cytoskeleton-like system, in which reversible polymerization/depolymerization of spiropyran/merocyanine-modified peptide nanofiber in giant unilamellar vesicles (GUVs) dramatically changed the morphology of GUVs with diameters that are near cell size [Fig. 4(c)].^61^ Spiropyran-modified peptide FKFEC^SP^KFE formed β-sheet nanofibers, whereas merocyanine-form photoisomerization completely dissociated the nanofibers. Spherical GUVs that encapsulate the merocyanine-peptide FKFEC^MC^KFE dramatically changed into worm-like vesicles by the photoisomerization to the spiropyran-form. UV and visible light irradiation reversibly controlled the dramatic morphological changes in GUVs. This photo-controlled system, which mimics the polymerization and depolymerization of the natural cytoskeleton, can be used as a component of molecular robots and artificial cell systems.

Moreover, several studies focused on photocontrol of self-assembly of peptides modified with various photoswitches. Poloni *et al.* revealed that photoisomerization of tryptophan zipper peptide modified with overcrowded alkene photoswitch generated two thermally stable isomers and controlled the formation of amyloid-like fibrils via β-hairpin structure.^62^ Marafon *et al.* developed photoisomerizable unsaturated β-amino acid, (*Z/E*)-3-aminoprop-2-enoic acid, to enable photo-control of self-assembly of peptide fordmars.^63^ Nakamura *et al.* showed the photo-triggered out-of-equilibrium pattern formation using peptide-type nanofiber modified with benzoylhydrazone group as photoswitch in self-sorting double network hydrogel.^64^ Light irradiation using a photomask followed by thermal incubation induced the spatially controlled peptide nanofiber condensation via metastable nanofiber photodecomposition in the irradiated areas and supply of monomers from the nonirradiated areas. Sun *et al.* developed a light-triggered platform that allows spatiotemporal control of self-assembly from nanoparticles into nanofibers in living cells for controlling cellular behaviors.^65^ A photoswitch 3-methylene-2-(quinolin-8-yl) isoindolin-1-one was modified with a peptide having an integrin binding sequence (RGD) and self-assembling sequence (SGKLVFF). Peptide conjugate photoisomerization caused a morphological change of the peptide assemblies on the cell surface from nanoparticles to nanofibers, which induced cell adhesion. Ji *et al.* showed that the morphology and gel properties of diphenylalanine assemblies can be modulated by light through coassembly with photochromic bipyridine derivatives.^66,67^

As described above, structural control of peptide assemblies by photoisomerization has greatly helped not only in controlling biological functions but also in developing novel drug delivery systems and bottom-up nanotechnology.

### B. Peptide–DNA interactions controlled by photoisomerization

**FIG. 5.**
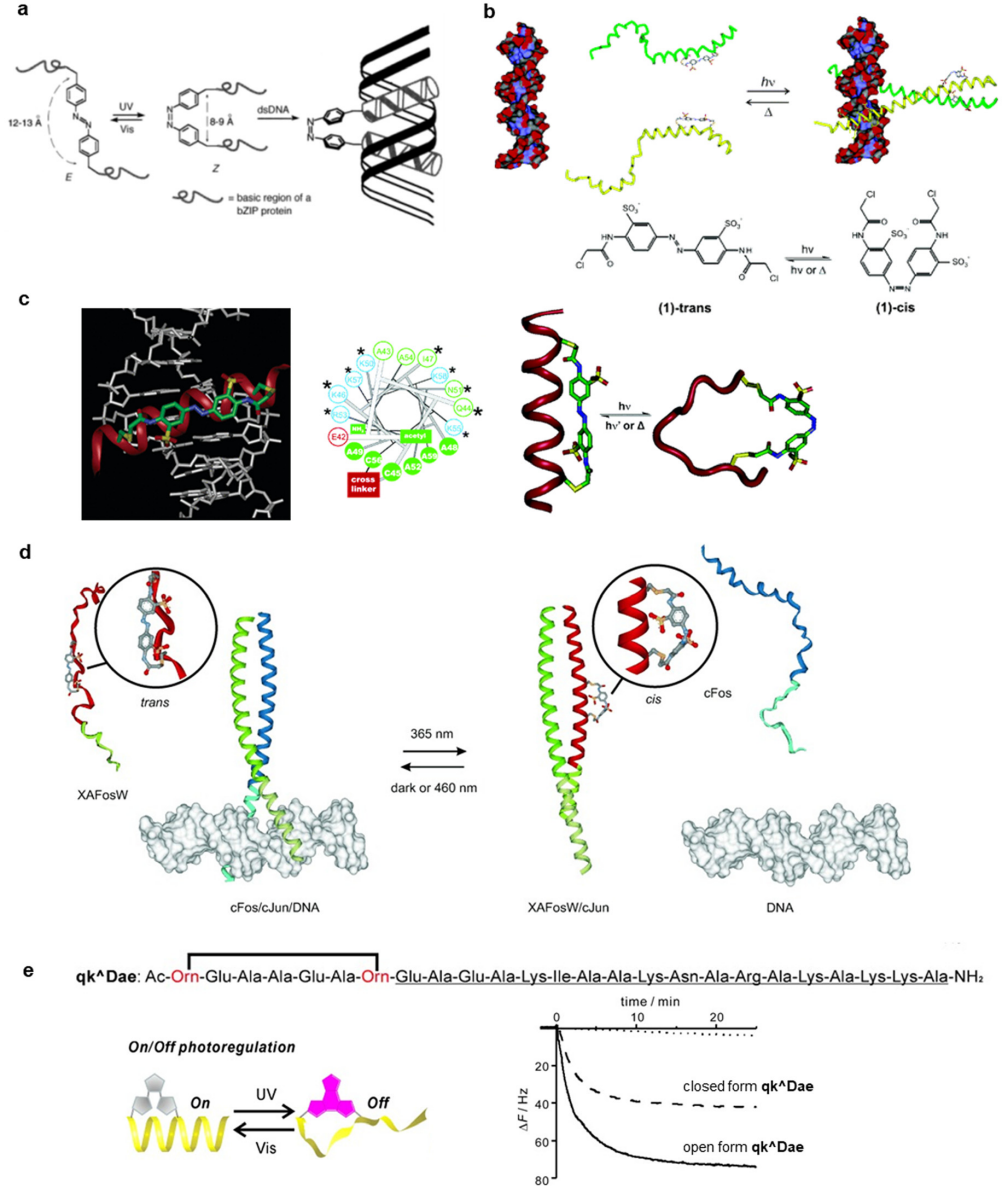
Peptide-DNA interactions controlled by photoisomerization. (a) Photocontrol of DNA-binding of two basic regions of bZIP protein linked through azobenzene. Reproduced with permission from Caamaño *et al.*, Angew. Chem. Int. Ed. **39**, 3104–3107 (2000). Copyright 2000 Wiley-VCH. (b) Reversible photocontrol of DNA binding of azobenzene-bridged GCN4-bZIP. Reproduced with permission from Woolley *et al.*, Biochemistry **45**, 6075–6084 (2006). Copyright 2006 American Chemical Society. (c) DNA-binding photocontrol of azobenzene-bridged miniature helix peptide. Reproduced with permission from Guerrero *et al.*, J. Am. Chem. Soc. **127**, 15624–15629 (2005). Copyright 2005 American Chemical Society. (d) DNA-binding photocontrol of azobenzene-bridged coiled-coil proteins in living cells. Reproduced with permission from Zhang *et al.*, Angew. Chem. Int. Ed. **49**, 3943–3946 (2010). Copyright 2010 Wiley-VCH. (e) Diarylethene-bridged helix peptides to photoregulate DNA-binding. QCM sensorgrams of diarylethene-bridged helix peptides binding to DNA immobilized on plates. Reproduced with permission from Fujimoto *et al.*, Chem. Eur. J. **18**, 9834–9840 (2012). Copyright 2012 Wiley-VCH.

Peptide–DNA interactions are abundant in living cells, and controlling these interactions allows the control of various intracellular signals such as gene expression. Sequence-specific DNA-binding proteins, known as transcription factors, primarily regulate gene expression in eukaryotes. Basic leucine zipper (bZIP) transcription factors, such as yeast GCN4, are dimeric proteins that recognize dyadic and mostly palindromic DNA sites,^68^ and studies investigated the control of GCN4-bZIP binding to DNA by various external stimuli.^69–73^ Caamaño *et al.* developed a peptide conjugate, wherein two basic regions of bZIP protein were linked through azobenzene as photoswitch [Fig. 5(a)].^74^ The *cis* isomer bound to duplex DNA containing the recognition site with a high affinity of 60–70 times more efficiently than the *trans* isomer, due to the geometric proximity of peptides. CD spectra revealed an increased α-helix content of the *cis* isomer upon DNA binding, whereas the ellipticity increase was smaller for the *trans* isomer. However, the reversibility of DNA binding was incomplete, because the DNA binding inhibits the *cis*-to-*trans* photoisomerization and thermal isomerization process. Woolley *et al.* developed a GCN4-bZIP bridged with an azobenzene between Cys residues within the zipper domain to achieve the reversible photo-control of DNA-binding of GCN4-bZIP [Fig. 4(b)].^75^ The *trans*-form destabilizes the helical structure of the coiled-coil region of GCN4-bZIP, whereas photoisomerization to the *cis*-form increases helical content and substantially enhances DNA binding. Since the thermal relaxation to the *trans* state and concomitant dissociation of the protein−DNA complex readily occurred, the photo-control of DNA-binding was observed to reversible. Furthermore, Guerrero *et al.* achieved photo-control of DNA-binding of HDH-3, an 18-residue miniature engrailed homeodomain, by cross-linking azobenzene through two Cys residues [Fig. 4(c)].^76^ The *trans-*form of the azobenzene-bridged HDH-3 bound to the target DNA with higher affinity (*K_d_* = 7.5 nM) than the *cis*-form after UV irradiation (*K_d_* = 140 nM). The remarkable affinity of the *trans-*form with only 18 amino acid residues was most likely a consequence of the cross-linker-induced preorganization of its recognition helix.

The reversible DNA-binding photocontrol could pave the way for studies of cellular processes through transcription control. Zhang *et al.* revealed that the introduction of a designed azobenzene-bridged dominant negative peptide (XAFosW) controlled the activity of a coiled-coil protein, the AP-1 transcription factor, in living cells [Fig. 4(d)].^77^ The photoisomerization to the *cis*-form enhanced helicity and induced strand exchange of coiled-coil to dissociate AP-1 transcription factor (cFos/cJun) from DNA. The AP-1 activity in living cells was inhibited with the XAFosW based on UV light irradiation.

Photocontrol using other DNA-binding motifs is being considered. Murawska *et al.* developed an AT-hook binding motif peptide incorporated azobenzene linker as a minor groove binder and a zink-finger peptide incorporated azobenzene linker as a major groove binder.^78^ Photochemical isomerization in each case affected DNA binding, as determined in fluorescent displacement assays on model DNA strands, which provides promising tools for DNA modulation. Photochromic molecules, other than azobenzene, have also been used for peptide-DNA interaction photocontrol. Azobenzenes have an undesirable property for biological use, such as thermal isomerization between the two photoisomers, but diarylethene never isomerizes without photoirradiation. Fujimoto *et al.* developed peptides possessing diarylethene-bridged and DNA-binding regions and achieved DNA-binding photocontrol without thermal isomerization [Fig. 4(e)].^79^ The open-form of the diarylethene-bridged peptide qk̂Dae folded into stable α-helices, whereas the closed-form after UV irradiation destabilized the helical structures. Quartz-crystal microbalance (QCM) analysis revealed that the open isomer is strongly associated with a target DNA compared with the closed one.

### C. Peptide–protein interactions controlled by photoisomerization

Specific peptides have a great affinity to target proteins to inhibit or activate their activities. Photochromic molecule conjugation to the ligand peptides is a promising approach to photocontrol target protein activities by changing the interactions between the peptides and proteins. Two groups (Liu *et al.*^80^ and Hamachi *et al.*^81^) developed the concept using ribonuclease S, an RNA hydrolyzing enzyme, which consists of S-peptide and S-protein fragments. The reconstitution efficiency of the mutated S-peptide to S-protein changed in the *cis*- and *trans*-states by incorporating an azobenzene moiety to S-peptide, thereby enabling the reversible photocontrol of enzymatic activity of the reconstituted ribonuclease S by light irradiation.^81^ Currently, incorporating photochromic molecules can control various types of protein activities, as shown below.

**FIG. 6.**
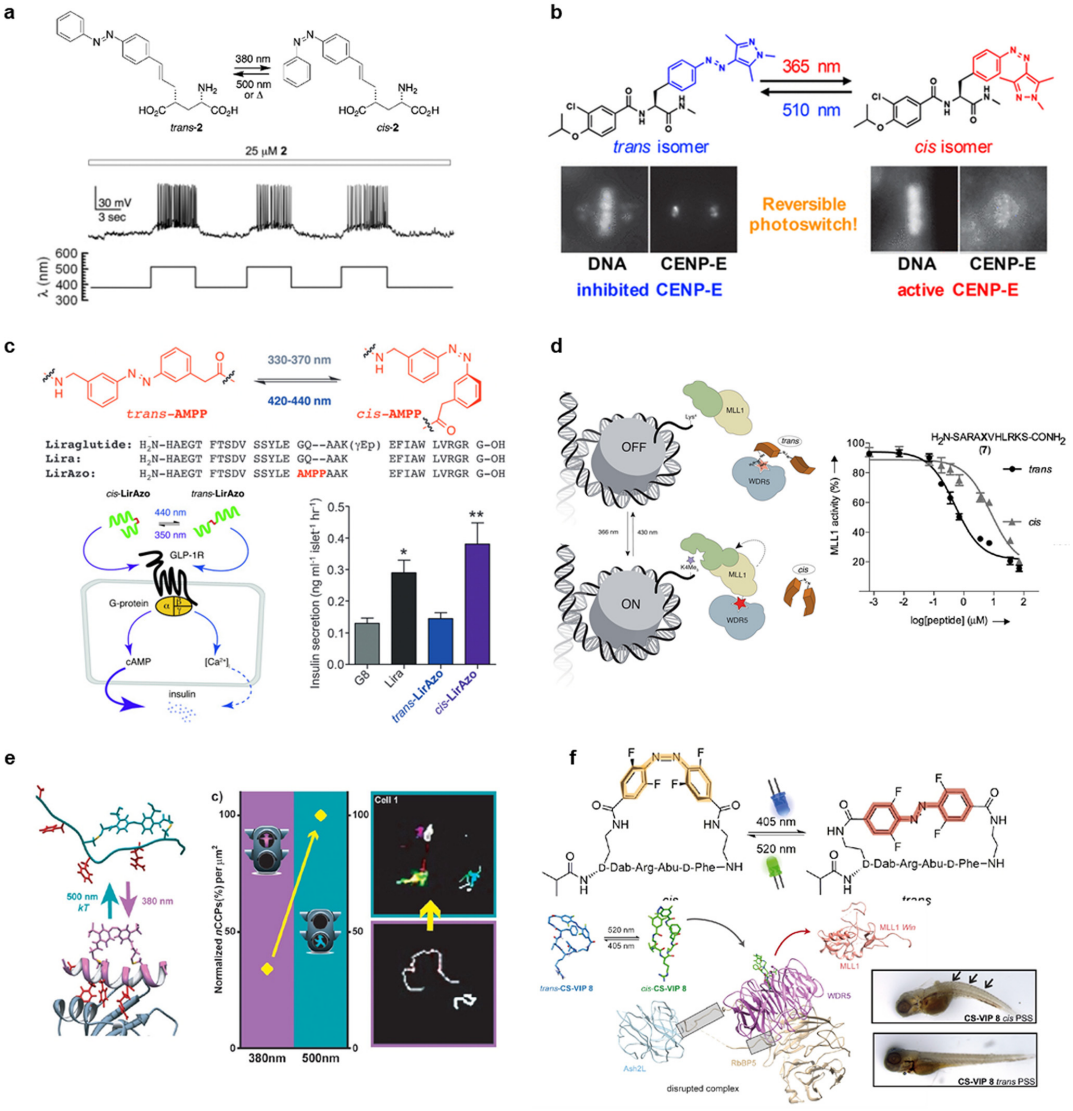
Photoisomerization controls peptide-protein interactions. (a) Photochromic glutamate analog for glutamate receptor agonist and whole-cell current clamp recording. Reproduced with permission from Volgraf *et al.*, J. Am. Chem. Soc. **129**, 260 − 261 (2007). Copyright 2007 American Chemical Society. (b) Photoswitchable CEMP-E inhibitor (a mitotic kinesin) regulating mitotic progression. Reproduced with permission from Mafy *et al.*, J. Am. Chem. Soc. **142**, 1763 − 1767 (2020). Copyright 2020 American Chemical Society. (c) Insulin secretion control by a photoswitchable incretin. Reproduced with permission from Broichhagen *et al.*, Angew. Chem. Int. Ed. **54**, 15565–15569 (2015). Copyright 2015 Wiley-VCH. (d) Photoswitchable MLL1-WDR5 interaction inhibitors control MLL1 methyltransferase activity. Reproduced with permission from Albert *et al.*, Chem. Sci. **8**, 4612–4618 (2017). Copyright 2017 Royal Society of Chemistry. (e) Photoswitching peptide inhibitors to β-appendage of AP2 regulating clathrin-mediated endocytosis. Reproduced with permission from Nevola *et al.*, Angew. Chem. Int. Ed. **52**, 7704 − 7708 (2013). Copyright 2013 Wiley-VCH. (f) Photoswitchable histone methyltransferase MLL1 inhibitor controlling hematopoiesis in zebrafish. Reproduced with permission from Albert *et al.*, ACS Cent. Sci. **8**, 57−66 (2022). Copyright 2022 American Chemical Society.

Volgraf *et al.* developed azobenzene-conjugated peptide agonists of ionotropic glutamate receptors that are crucial mediators of excitatory information transfer in the central nervous system to photocontrol the ion channel activities.^82,83^ In their first approach, the azobenzene-conjugated agonist covalently tethered to the mutated cysteine residue of the target ionotropic glutamate receptor, iGluR6, to open/close the channel,^82^ as a similar strategy to photocontrol the potassium channel.^84^ In their second approach, non-tethered azobenzene-conjugated agonists that can be used for wild-type ionotropic glutamate receptors, iGluR5 and iGluR6, were developed [Fig. 6(a)].^83^ The azobenzene-conjugated glutamate analog showed channel activation in the *trans*-form compared to the *cis*-form in the whole-cell voltage clamp assay in HEK293 cells that express iGluR6. Further, the system was used to reversibly activate iGluR6 channels in cultured rat hippocampal neurons by switching wavelengths of light between 380 and 500 nm [Fig. 6(a)]. Mafy *et al.* developed an azobenzene-conjugated peptide inhibitor of centromere-associated protein E (CENP-E), which is a mitotic kinesin used for chromosome transportation [Fig. 6(b)].^85,86^ The design was based on the known CENP-E inhibitor that prevents chromosome alignment and mitotic progression. The azobenzene-conjugated peptide inhibitor showed ∼10-fold higher inhibition CENP-E activity in the *trans*-form compared to the *cis*-form. This system was used to reversibly photocontrol the CENP-E-dependent chromosome congression and mitotic progression by switching light wavelengths (365 and 510 nm). Not only the azobenzene moiety but also the thioxylation (O/S exchange) of the peptide bond could be used for the *cis*- and *trans*-photoisomerization toward photocontrol of the binding to target proteins.^87^

The introduction of photochromic molecules to the backbone of peptides is an attractive approach for inducing dynamic structural changes in peptides. Azobenzene-based backbones were frequently used for this purpose due to their synthetic simplicity and commercial availability. Hodson and Trauner developed incretin mimetics that contain the azobenzene backbone to photocontrol the binding to the target glucagon-like peptide-1 receptor (GLP-1R) resulting in insulin secretion [Fig. 6(c)].^88^ They introduced azobenzene moiety as a bridge between two α-helixes of the original GLP-1R agonist and evaluated the effects of the peptide (LirAzo) on the cellular GLP-1R signaling of Ca^2+^ and cyclic AMP (cAMP) followed by insulin secretion. The *trans*-LirAzo induced the increment of Ca^2+^ influx compared to *cis*-LirAzo, whereas *cis*-LirAzo induced cAMP generation compared to *trans*-LirAzo. With different signaling, *cis*-LirAzo augmented glucose-induced insulin secretion compared to *trans*-LirAzo, whereas *trans*-LirAzo demonstrated a significant protective effect against glucolipotoxicity compared to *cis*-LirAzo. The azobenzene backbone strategy was used for photoswitchable inhibition of mixed-lineage leukemia 1 (MLL1) methyltransferase core complex [Fig. 6(d)].^89^ The azobenzene-conjugated peptide inhibitors were designed for the photocontrol of the methylation activity because the binding of MLL1 and WD40-repeat protein 5 (WDR5) is crucial for the methylation activity. A peptide that demonstrated a higher inhibition activity in the *trans*-form compared to the *cis*-form and the original WDR5-interacting peptide was found by optimizing the position of the azobenzene that was introduced to the WDR5-interacting peptide. The *in vitro* assay showed that the *trans*-form demonstrated a stronger MLL1 methyltransferase activity inhibition compared to the *cis*-form [Fig. 6(d)]. The system was used for suppressing MLL1-target gene transcription and inhibiting leukemia cell growth by incorporating octa-arginine (a common cell-penetrating peptide) into the peptide. In another example, reversible photocontrol of cell adhesion was achieved by introducing an azobenzene backbone between a cell adhesive RGD peptide and alkanethiols that was used for substrate immobilization.^90^

Light-driven change of secondary peptide structures is also useful to control the interaction with target proteins. Hence, stapled peptides that are linked by azobenzene moieties were developed. Kneissl *et al.* designed α-helical peptides stapled with azobenzene moiety for photoswitching the secondary peptide structures by *cis*- and *trans*-isomerization that are targeted to an anti-apoptotic protein, Bcl-x_L_.^91^ Nevola *et al.* used a similar strategy for the photocontrol of protein–protein interaction of the AP2 complex that is involved in the clathrin-mediated endocytosis [Fig. 6(e)].^92^ The design was based on the C-terminal β-arrestin peptide that binds to the β-appendage of AP2 with an α-helical structure. Several peptides that adopt a helical structure and bind to β-appendage of AP2, preferably in the *trans*-form or *cis*-form were obtained, by stapling the peptide using an azobenzene crosslinker by the reaction with cysteine residues that were introduced at different positions. These peptides provided “stop” and “go” signals to the clathrin-mediated endocytosis by photocontrolling the binding to β-appendage of AP2. In particular, one azobenzene-stapled peptide inhibited the clathrin-mediated endocytosis of transferrin efficiently in the *trans*-form by slowing down the membrane traffic, as observed in the light-dependent increased/decreased numbers of clathrin-coated pits [Fig. 6(e)]. The azobenzene-stapled peptide strategy was also used to photocontrol the interaction between S-peptide and S-protein.^93^ Azobenzene-conjugated cyclic peptides were recently developed as photoswitching inhibitors of the histone methyltransferase MLL1 complex that has various hematopoietic roles, such as a chromatin-modifier and a potent oncogenic driver toward hematopoietic control in zebrafish [Fig. 6(f)].^94^ The design was based on a cyclic peptide, MM-401, the potent inhibitor of MLL1 complex, including MLL1, WDR5, RbBP5, Ash2L, and DPY30. One of the designed azobenzene-tethered peptides strongly bound to WDR5 as a potential MLL1 methylation activity inhibitor in the *cis*-form than the *trans*-form. This system was used to photocontrol the hematopoiesis of zebrafish. Peptide treatment in the *cis*-form resulted in a lack of responsivity upon tail touch in the larvae and induced abnormal developmental phenotypes (curved body axis and heart edema), whereas the *trans*-form demonstrated no apparent effects [Fig. 6(f)]. Additionally, larvae incubation with the *trans*-form followed by light irradiation (520 nm for 30 s) and subsequent incubation for 18 h showed comparable effects to the *cis*-form treatment. Another unique approach is the cross-linking of two enzyme sites using azobenzene moiety to photocontrol the enzymatic activity. Schierling *et al.* utilized this approach to photocontrol the activity of the homodimeric restriction endonuclease PvuI, where the dramatic activity change was observed by introducing two azobenzene moieties.^95^

These examples demonstrated that incorporating photochromic molecules into peptides is a promising approach to photocontrol peptide interactions to target proteins *in vitro* and *in vivo*.

### D. Cytoskeleton functions controlled by photoisomerization

Cytoskeletons, such as microtubules and actin filaments, are crucial cell components. Controlling the structures and functions of the cytoskeletons by light is a promising approach for manipulating cell functions because of their important functions in cells, such as structural support, cell division, cell migration, and intracellular transport. The major strategy of controlling the cytoskeletons by light is photochromic molecule conjugation to the cytoskeleton-targeted drugs or the design of photochromic molecules mimicking the drugs, which was developed by Borowiak *et al.* Their first report designed photostatins, which show reversible *cis*–*trans* isomerization by blue and green light, based on a microtubule-destabilizing drug, colchicine.^96^ Photostatins show a stronger microtubule-disrupting effect in *cis*-state compared to *trans*-state, which can be used to control microtubule dynamics in cells. They later reported the detailed analysis and *in vivo* applications of photostatins.^97^ Afterward, various types of photoswitches were developed for microtubule^98–106^ and actin filament control,^107–109^ mainly by photochromic molecule conjugation such as azobenzenes to the cytoskeleton-targeted drugs. Additionally, a tubulin-specific photoswitchable fluorescent probe was developed by conjugating the spiropyran moiety to colchicine unit.^110^

The cytoskeletons, in addition to cellular applications, can be used as components of dynamic nanomaterials. Microtubules are frequently used for combining with motor proteins (kinesin and dynein) to construct ATP-driven motile nanomaterials. The control of movement, stability, and assembling properties of microtubules are important for constructing sophisticated motile systems. Keya *et al.* developed light-responsive control of microtubule swarming by conjugating photoresponsive DNA-containing azobenzene groups to the outer microtubule surface.^111^ In this case, microtubule swarm formation was promoted in the *trans*-state and inhibited in the *cis*-state of the azobenzene moiety. New types of mechanical robots include the reversible association/dissociation of microtubule swarms by light. Later, the mechanical properties of microtubules^112^ and cargo delivery^113^ were controlled using the photoresponsive DNA-conjugated microtubules.

**FIG. 7.**
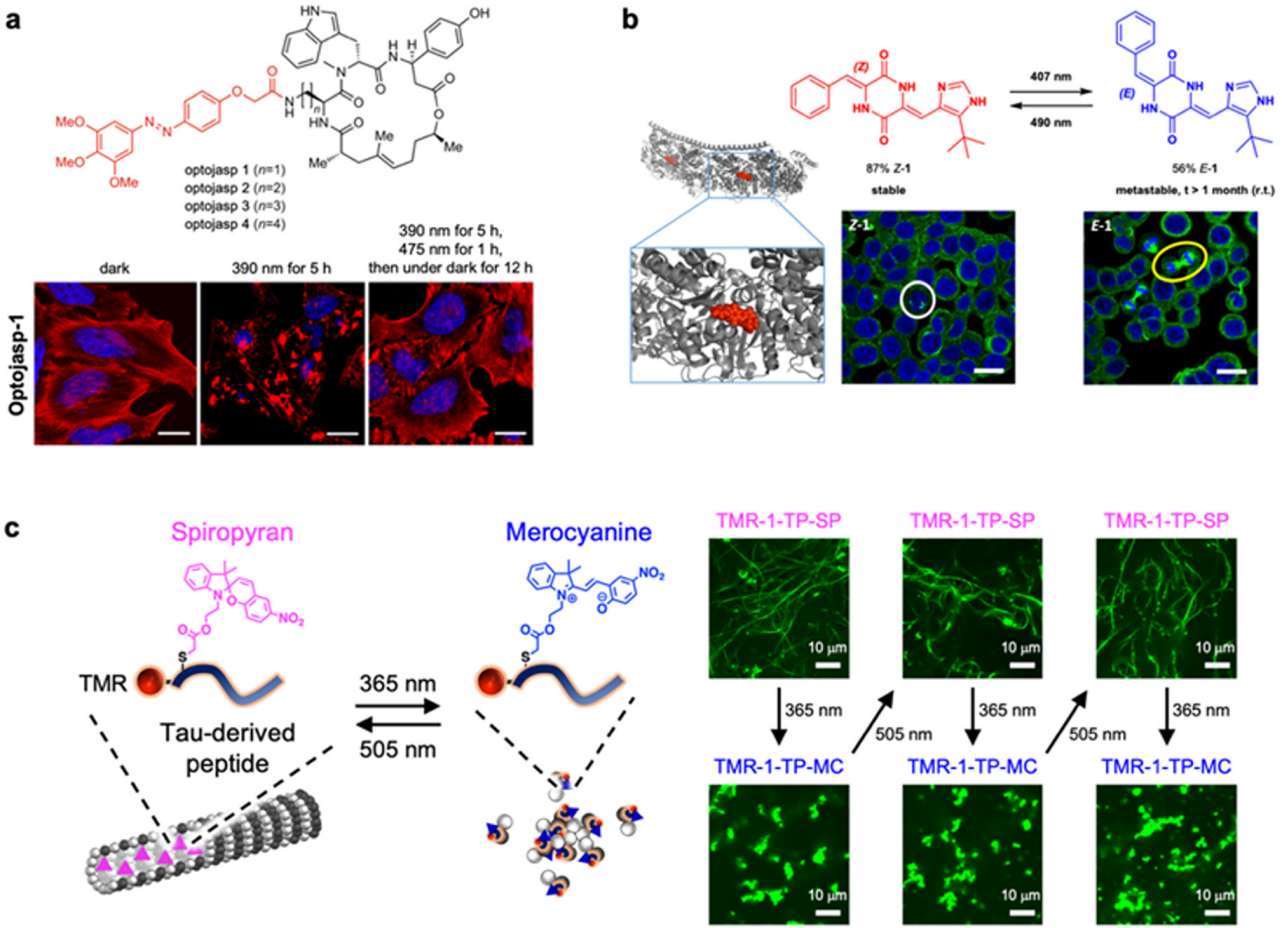
Control of cytoskeletons by photochromic peptides. (a) Control of actin filament dynamics by azobenzene-conjugated depsipeptide. Reproduced with permission from Borowiak *et al.*, J. Am. Chem. Soc. **142**, 9240 − 9249 (2020). Copyright 2020 American Chemical Society. (b) Peptide-based photoswitch for the control of microtubule dynamics. Reproduced with permission from Kirchner *et al.*, Nat. Commun. **13**, 6066 (2022). Copyright 2022 Springer Nature. (c) Spiropyran-conjugated TP reversibly photocontrol microtubule structures. Reproduced with permission from Inaba *et al.*, ChemBioChem **24**, e202200782 (2023). Copyright 2023 Wiley-VCH.

Recently, peptides that are conjugated with photoisomerization moiety were developed to control the structures and dynamics of actin filaments and microtubules. Borowiak *et al.* developed light-dependent actin filament effectors by conjugating azobenzene to actin filament-stabilizing marine depsipeptide jasplakinolide [Fig. 7(a)].^107^ Among the designed molecules, termed optojasps, optojasp-1 with the shortest linker demonstrated cytotoxicity in *cis*-form compared to *trans*-form. Additionally, the *cis*-form of optojasp-1 increased actin nucleation and caused large aggregate formation, such as actin filament stabilizer, including phalloidin, whereas the *trans*-form demonstrated no such effect. The irradiation of 390 nm of light to the optojasp-1-treated cells in the *trans*-state for 5 h caused actin filament aggregation [Fig. 7(a), bottom left]. The irradiation of 390 nm of light and the subsequent irradiation of 475 nm of light for 1 h followed by incubation under a dark environment for 12 h recovered the regular actin filament structures [Fig. 7(a), bottom right]. Thus, these structural changes of actin filaments were reversibly controlled by light irradiation. This system was also used to photocontrol the cellular dynamics related to actin filaments, including cell shapes, motility, and division. Later, the cryo-EM structures of actin filaments with optojasps were obtained.^108^ Additionally, new types of optojasps that can be activated with longer wavelengths in the visible range (e.g., 440–477 nm) were developed.^109^ These molecular tools help understand and control the dynamics of actin filaments.

Kirchner *et al.* used a peptide-derived photoswitchable motif, plinabulin (a tubulin polymerization inhibitor), as a photochromic molecule to optically control microtubules [Fig. 7(b)].^114^ Plinabulin consists of a cyclic dipeptide core with the arylidene residue [Fig. 7(b)]. They focused on the use of plinabulin as a photochromic molecule based on the *E/Z*-photoisomerization of arylidene substituents in other examples. The reversible *E/Z*-photoisomerization of plinabulin was observed, which revealed the dramatically different cytotoxicity against HT-29 human colon cancer cells between *Z*-form (thermodynamically stable form, IC_50_ = 0.47 nM) and *E*-form (metastable form, IC_50_ = 92 nM). The *Z*-form of plinabulin induced abnormal tubulin aggregation and mitotic arrest in several cells, whereas the *E*-form demonstrated regular cell division as mitotic spindles were observed. The derivatives and the shorter version of plinabulin showed different light sensitivity, reversibility, and cytotoxicity, indicating that the chemical structure modification is useful to optically controlling microtubule dynamics leading to cell manipulation.

Spiropyran was conjugated to Tau-derived peptide (TP), which was bound to the inner pocket of microtubules, to reversibly alter microtubule configurations by light [Fig. 7(c)].^115^ Spiropyran was conjugated to the cysteine residues that were introduced to the different TMR-labeled TP positions. The peptide with spiropyran at the N-terminus (TMR-1-TP-SP) and the merocyanine form (TMR-1-TP-MC), among the four peptides with different positions of spiropyran, showed the largest difference in microtubule structures prepared with GTP, as TMR-1-TP-SP stabilized the microtubule structures but not TMR-1-TP-MC [Fig. 7(c)]. The structural change of microtubules by TMR-1-TP-SP/TMR-1-TP-MC was reversible and repetitive by UV light (365 nm) and visible light (505 nm) irradiation. The photocontrol of microtubule structures using a reversible photoisomerization system is useful for the spatiotemporal manipulation of cell fates.

## III. PHOTOCLEAVABLE PEPTIDE MATERIALS

### A. Design of photocleavable peptides

Photocleavage reactions, including deprotection of the protecting group and cleavage of the peptide backbone, are a powerful tool to activate or deactivate functional peptides and proteins with high spatiotemporal resolution. Most photocleavage of peptide backbones is based on intramolecular cyclization of the photoexcited side chain group followed by amide bond cleavage [Fig. 2(b)]. Peptide backbone that incorporated 2-nitrophenylalanine and developed by Peters *et al.* is readily cleaved by UV irradiation via intramolecular cyclization.^116^ Furthermore, they revealed that genetically engineered protein containing 2-nitrophenylalanine was site-specifically photocleaved. Taniguchi *et al.* developed a photo-triggered production system of Aβ1 − 42 via photocleavage of the protecting group (6-nitroveratryloxycarbonyl) followed by O−N intramolecular acyl migration.^117^ Katayama *et al.* created photocleavable peptides with a bromocoumarin linker having a high photolytic efficiency.^118^ Shigenaga *et al.* developed photocleavable peptides that can release a functional peptide after the stimulus-induced removal of a phenolic protective group and subsequent cleavage of peptide backbone through nucleophilic attack of phenolic hydroxyl group to an adjacent peptide bond.^119–121^ The system was used for the photo-responding release of nucleocytoplasmic shuttle peptide to control intracellular localization with light.^119^ Mangubat-Medina *et al.* developed a new photocaging method using histidine-directed backbone modification to selectively modify peptides and proteins at the amide N–H bond, which allows the photorelease of the backbone modification and function restoration.^122^

### B. Peptide assemblies induced by photocleavage

**FIG. 8.**
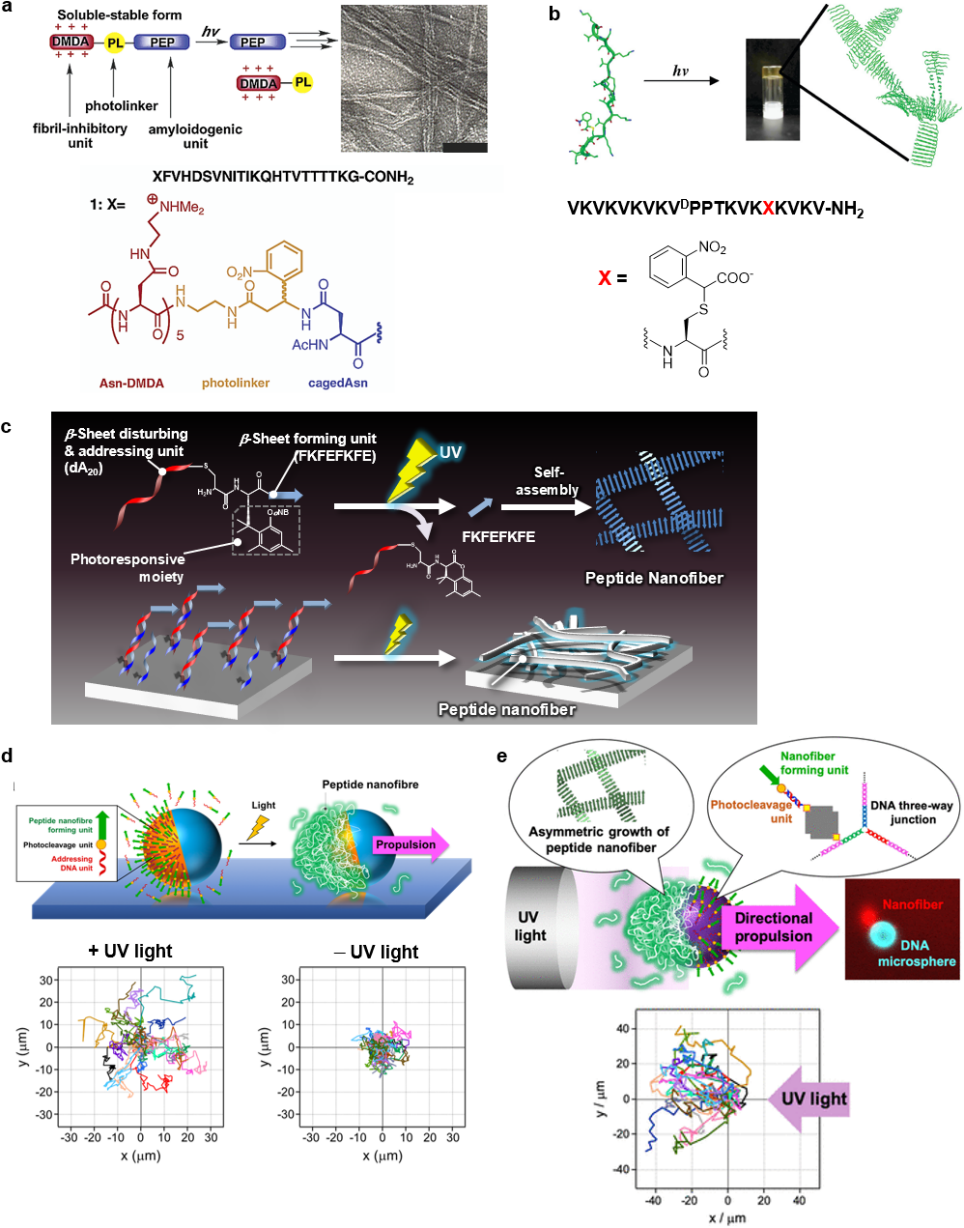
Peptide assemblies induced by photocleavage. (a) Temporal control of peptide fibrillization by photocleavage. Reproduced with permission from Bosques and Imperiali, J. Am. Chem. Soc. **125**, 7530–7531 (2003). Copyright 2003 American Chemical Society. (b) Light-activated hydrogel formation via β-hairpin peptide folding and self-assembly triggered by the photo-deprotection. Reproduced with permission from Haines *et al.*, J. Am. Chem. Soc. **127**, 17025–17029 (2005). Copyright 2005 American Chemical Society. (c) Spatiotemporal control of peptide nanofiber growth triggered by photocleavage of a DNA-conjugated β-sheet forming peptide. Reproduced with permission from Furutani *et al.*, Chem. Commun. **51**, 8020–8022 (2015). Copyright 2015 The Royal Society of Chemistry. (d) Light-induced propulsion of a giant liposome driven by peptide nanofibre growth. The tracking trajectories of giant liposomes by UV light irradiation. Reproduced with permission from Inaba *et al.*, Sci. Rep. **8**, 6243 (2018). Copyright 2018 Authors, licensed under a Creative Commons Attribution (CC BY) License. (e) Light-induced peptide nanofiber growth derived negative phototaxis of nucleo-spheres. The tracking trajectories of nucleo-spheres by UV light irradiation. Reproduced with permission from Inaba *et al.*, ACS Appl. Bio Mater. **4**, 5425–5434 (2021). Copyright 2021 American Chemical Society.

Photocleavage^123–128^ of the peptide backbone or photodeprotection^129–133^ from the side chain described in Sec. III A can trigger spatiotemporal-controlled peptide self-assembly. Photo-induced elimination of units that inhibit the self-assembly of peptides can initiate the self-assembly with controlled timing. Bosques and Imperiali pioneered such a photo-induced peptide assembling system using peptide having a fibril inhibitory unit and amyloidogenic unit linked by a photocleavable amino acid [Fig. 8(a)].^123^ The peptide was not assembled due to electrostatic repulsion among polycationic *N,N*-dimethylethylenediamine units, while afforded fibrous assemblies after photolysis and subsequent incubation for several hours. Haines *et al.* developed a light-activated peptide hydrogel formation system using peptide photo-deprotection [Fig. 8(b)].^129^ MAX7CNB, a 20-residue peptide, is protected with α-carboxy-2-nitrobenzyl group at *Cys* residue and did not self-assemble due to the unfold conformation. UV irradiation released the protecting group and triggered peptide folding to produce amphiphilic β-hairpins that self-assemble into viscoelastic hydrogel material. The formed gel surface was non-cytotoxic for NIH 3T3 fibroblast cells, conducive to cell adhesion, and allowed cell migration. Recently, Xiang *et al.* developed a novel photo-triggered peptide hydrogel photocaged by a positively charged dipeptide.^124^ The photoactivated hydrogel was suitable for two-dimensional (2D) and 3D cell cultures, and its photo-controllable mechanical strength could regulate the spreading of stem cells on its surface.

We developed a spatiotemporal control system of peptide nanofiber growth by photocleavage of a DNA-conjugated β-sheet forming peptide linked through a photoresponsive amino acid residue [Fig. 8(c)].^125^ Single-strand DNA (dA_20_) was used not only as the β-sheet disturbing unit because of its electrostatic repulsion but also addressing unit on complementary dT_20_-immobilized materials by its hybridization. UV light irradiation to the conjugate caused the cleavage of the peptide bond to generate the phenolic intermediate and subsequent intramolecular cyclization and then releases free FKFEFKFE peptide, which self-assembles to form nanofibers. The photo-triggered nanofiber growth selectively occurred on the complementary DNA (dT_20_)-immobilized glass substrate, while the nanofiber formation was minimally observed on the non-complementary DNA substrate even after UV irradiation. Afterward, we demonstrated that the photo-triggered peptide nanofiber growth on asymmetric giant liposomes promoted the translational motion, as a functional mimicking system of “actin comet tail” [Fig. 8(d)].^126^ An improved DNA-peptide conjugate that is photo-cleaved faster was immobilized onto phase-separated giant liposomes using streptavidin–biotin interaction and DNA hybridization. UV light irradiation greatly enhanced the translational movement of the conjugate–modified giant liposomes and the movement was sustained even after UV light irradiation ceased, whereas the translational motion was suppressed without UV light irradiation. Force generation due to peptide nanofiber growth and the Marangoni effect due to the surface tension gradient between the nanofiber-forming side and the opposite side are the possible driving forces of propulsion.

In nature, various phototactic microorganisms move toward (positive phototaxis) and away (negative phototaxis) from the light source. Recently, we demonstrated that DNA microspheres equipped with the photo-triggered nanofiber growth system showed negative phototaxis [Fig. 8(e)].^127^ DNA-peptide conjugate that was connected by a photocleavage site was immobilized on a biotin-modified nucleo-sphere that was self-assembled from DNA three-way junctions bearing self-complementary sticky-ends. UV light irradiation induced the asymmetric peptide nanofiber growth on the nucleo-sphere surface and the directional movement away from the light source. Peptide nanofiber growth could be induced only on the light-irradiated side and not the opposite side because the interior of the nucleo-spheres is filled with DNA and has a light-scattering property, resulting in negative phototaxis. The artificial phototactic system based on peptide nanofiber formation will not only be useful for understanding phototactic microorganisms in nature but may also result in future applications for microrobot construction.

### C. Biological functions controlled by photocleavage of peptides

Peptide photocleavage was used to photocontrol the biological functions, such as ligand binding to targets,^134–145^ cell adhesion/death,^146–152^ and intracellular localization.^153–155^ The photocleavage reaction is irreversible in contrast to the reversible structural change of photochromic molecules, but peptide cleavage possibly induces large structural changes. Examples of the photocleavage strategy to photocontrol the biological functions *in vitro* and *in vivo* are described below.

**FIG. 9.**
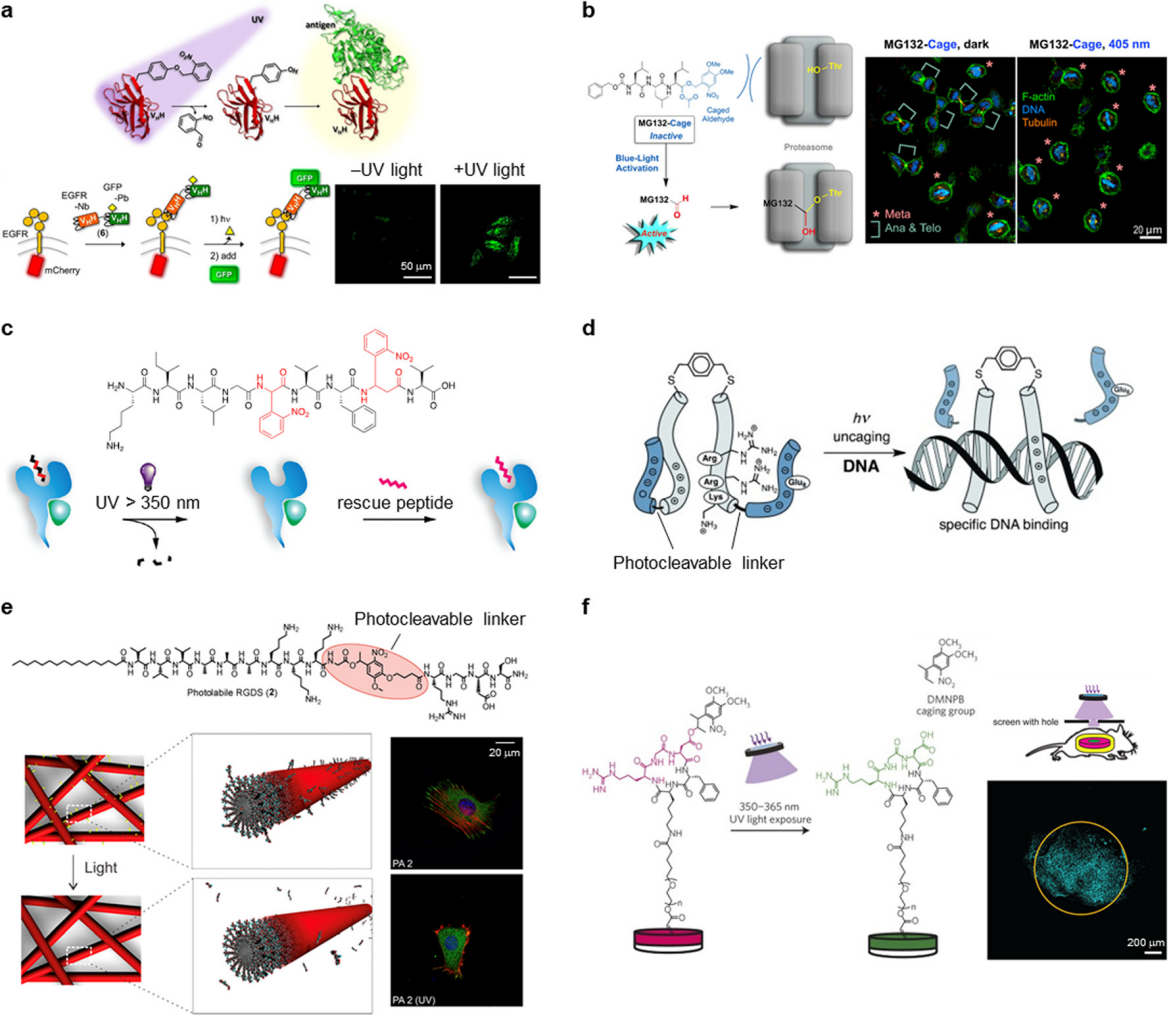
Peptide photocleavage controls biological functions. (a) Light-activatable binding of nanobody to its antigen. Reproduced with permission from Jedlitzke *et al.*, Angew. Chem. Int. Ed. **59**, 1506−1510 (2020). Copyright 2020 Wiley-VCH. (b) Light-activatable binding of covalent inhibitor, MG132, to proteasome and cell apoptosis. Reproduced with permission from Uhl *et al.*, Angew. Chem. Int. Ed. **60**, 1187−1196 (2021). Copyright 2021 Wiley-VCH. (c) Light-induced ligand exchange with a photocleavable peptide ligand. Reproduced with permission from Celie *et al.*, J. Am. Chem. Soc. **131**, 12298−12304 (2009). Copyright 2009 American Chemical Society. (d) Light-induced DNA binding of peptide dimer. Reproduced with permission from Jiménez-Balsa *et al.*, Angew. Chem. Int. Ed. **51**, 8825−8829 (2012). Copyright 2012 Wiley-VCH. (e) Photolytic RGDS peptide removal from peptide amphiphile nanofibers and effects on the cell adhesion. Reproduced with permission from Sur *et al.*, ACS Nano **6**, 10776−10785 (2012). Copyright 2012 American Chemical Society. (f) Light-induced activation of cell adhesion of caged cyclic RGD peptide on hydrogels and patterning of *in vivo* cell adhesion. Reproduced with permission from Lee *et al.*, Nat. Mater. **14**, 352−360 (2015). Copyright 2015 Springer Nature.

Incorporation of the photochromic moieties into the peptides is widely used to photocontrol the binding to target proteins. In particular, peptide ligands were caged with photocleavage moieties to decrease the binding affinity to target proteins, whereas the photocleavage reaction recovered the binding property to target proteins.^134,135^ In another approach, the peptide-based ligands with photocleavage moiety inhibited the activity of kinase^136^ and antibody,^137^ where the photocleavage reactions released the peptides and restored targeted protein activities. Additionally, the photocleavage technique was used to analyze the binding kinetics of peptides to target proteins.^138^ A similar concept was used to construct photocaged antibody fragments (nanobody) to photocontrol the binding to the antigen [Fig. 9(a)].^139^ The photocaged tyrosine variants, in this example, were incorporated into the key residue for the binding to target GFP in an anti-GFP nanobody. Compared to the parent nanobody, the photocaged nanobody exhibited ∼10 000-fold impaired binding affinity. The light-induced binding of sfGFP to the photocaged nanobody in living cells was performed using this system [Fig. 9(a)]. The caging strategy was used for light-activated covalent inhibitors to photocontrol cell cycle and apoptosis [Fig. 9(b)].^140^ MG132, a covalent proteasome inhibitor peptide, was photocaged at the reactive aldehyde to inhibit the activity. Irradiation with 405 nm of light released the de-caged MG132 that bound to the proteasome, resulting in cell cycle arrest in metaphase and apoptosis [Fig. 9(b)]. The photocaging strategy of protein-binding peptides was used for other cellular applications, such as kinase photoactivation in living cells^141^ and enzymatic hydrogel patterning used for the 3D invasion of primary human mesenchymal stem cells.^142^

Photocleavable peptides were used for light-induced ligand exchange, mainly for ligand binding *in situ* analysis.^143,144^ In particular, the photocleavable peptide ligand was developed for analyzing the ligand binding to MHC class I protein crystals [Fig. 9(c)].^143^ UV light-induced cleavage of the peptide in the MHC class I protein crystals released the lytic fragment and allowed for new full-length peptide binding. The *in crystallo* ligand exchange could be used for the high-throughput structural determination of the protein–ligand complexes. Not only the binding of peptide ligands but also the binding of DNA was also controlled using the photocleavable peptides. The N-terminal positively charged region of DNA-binding GCN4 transcription factor that was connected to negatively charged eight Glu residues through a photocleavable amino acid was designed [Fig. 9(d)].^145^ The dimerized peptide demonstrated a weak binding to target DNA due to the electrostatic repulsion between the negatively charged Glu moiety and DNA. The Glu moiety was released and the DNA-binding property was restored upon UV light irradiation.

Cell adhesion photocontrol using the photocleavable peptides immobilized on the substrates was widely explored. One strategy includes light-induced cell detachment or decreased cell adhesion.^146–148^ Sur *et al.* developed self-assembling peptide amphiphiles connected with cell adhesion epitope RGDS through photocleavable units as a light-responsive synthetic matrix that mimics natural extracellular matrixes [Fig. 9(e)].^147^ The self-assembling nanofiber matrix demonstrated increased cell spreading and mature focal adhesion, whereas UV light treatment inhibited the cell spreading due to the release of RGDS peptides. The opposite strategy is the light-induced cell adhesion using caged RGD peptide on the substrate surface.^149–151^ Lee *et al.* used this strategy for *in vivo* control of cell adhesion [Fig. 9(f)].^151^ They designed poly(ethylene glycol) di-acrylate hydrogels that present caged cyclic RGD peptides where the carboxylic group of the Asp residue was photocaged. UV light-induced cell adhesion was observed due to active cyclic RGD peptide presentation using the hydrogels as substrates. Hydrogel implantation and transdermal UV light irradiation induced cell adhesion on the exposure spot [Fig. 9(f)], demonstrating the spatiotemporal control of cell adhesion *in vivo*. The immobilization of photocleavable peptides on the substrates was also used for peptide cytotoxicity analysis.^152^

## IV. PHOTOCROSSLINKING PEPTIDE MATERIALS

### A. Photocrosslinking of peptide assemblies

**FIG. 10.**
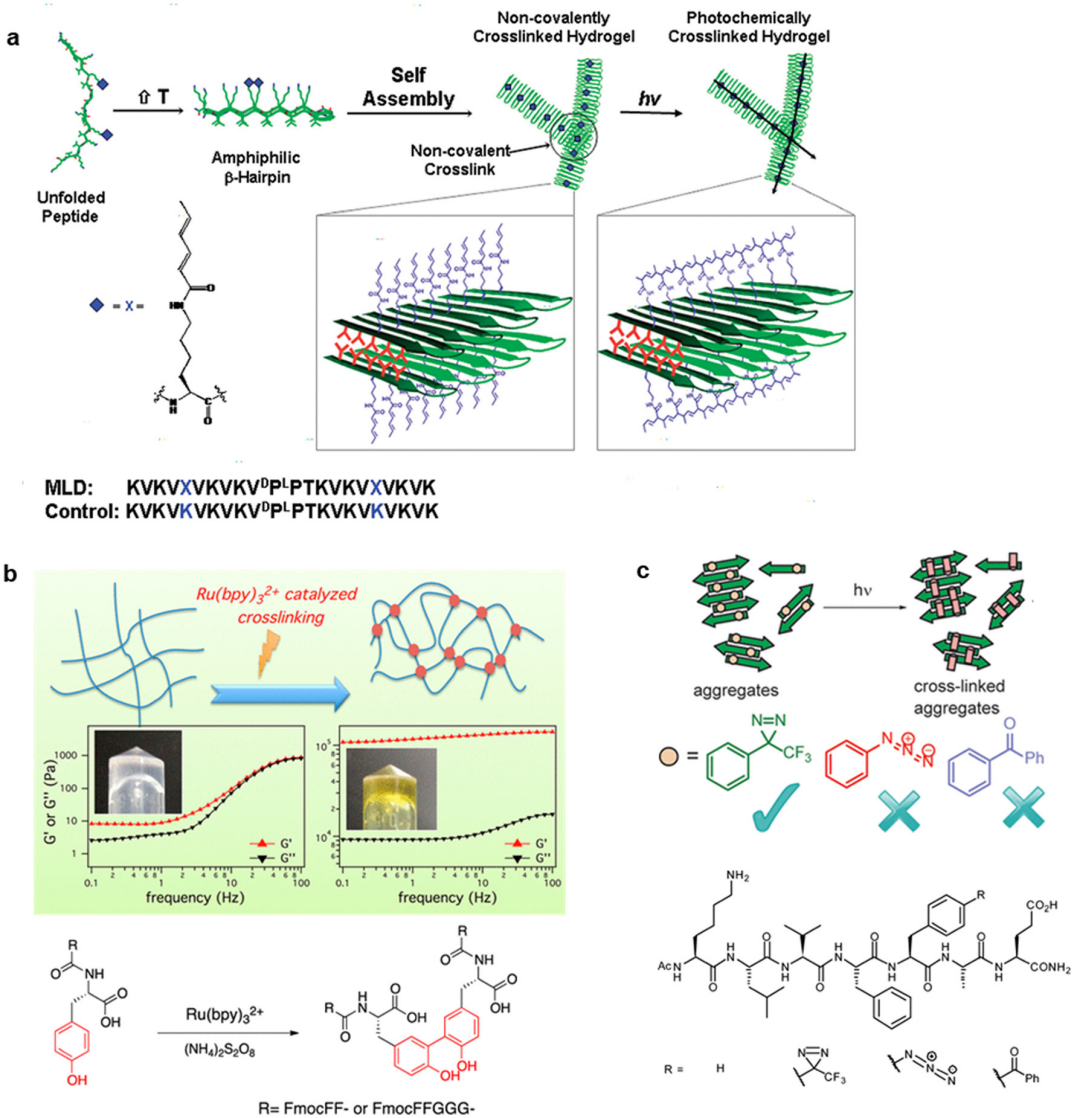
Photocrosslinking of peptide assemblies. (a) Intrafibrillar photopolymerization of β-hairpin peptide-based hydrogel. Reproduced with permission from Rughani *et al.*, Macromolecules **43**, 7924–7930 (2010). Copyright 2010 American Chemical Society. (b) Ruthenium-complex-catalyzed photocrosslinking of tyrosine-containing peptide hydrogels. Reproduced with permission from Ding *et al.*, Langmuir **29**, 13299–13306 (2013). Copyright 2013 American Chemical Society. (c) Photocrosslinking of amyloid-like nanostructures with diazirine derivative. Reproduced with permission from Preston *et al.*, ACS Chem. Biol. **9**, 761–768 (2014). Copyright 2014 American Chemical Society.

Photocrosslinking of peptides at the suitable position is expected to stabilize the secondary structure, promote self-assembly, and reinforce mechanical rigidity. Rughani *et al.* revealed that diene polymerization remarkably enhanced the mechanical rigidity of hydrogel self-assembled from an amphiphilic β-hairpin peptide that was modified with diene groups at the Lys side chain [Fig. 10(a)].^156^ Rheological experiments reveal that irradiation caused intrafibrillar cross-linking and a 2.5-fold increase in mechanical rigidity. This peptide hydrogel is expected to be used as an injectable material whose mechanical properties can be easily modulated post-delivery *in vivo*. Ding *et al.* revealed that ruthenium-complex-catalyzed photocrosslinking dramatically enhanced the mechanical stability of small tyrosine-containing peptide hydrogels by 10^4^-fold [Fig. 10(b)].^157^ The mechanical stability was enhanced due to the formation of a densely entangled fibrous network of peptide dimers through a dityrosine linkage. Peptide-based hydrogels frequently have low mechanical stability with storage moduli of 10–1000 Pa that greatly hinder their practical application, but the storage modulus of this crosslinked peptide hydrogel was approximately 100 kPa, which is one of the highest reported so far for hydrogels that are made of small peptide molecule. Recently, Pugliese *et al.* created a peptide hydrogel with pH-switchable on–off luminescence by ruthenium-complex-catalyzed photocrosslinking of a tyrosine-containing peptide, which is potentially applied for biomedical imaging, pH sensing, photonics, soft electronics, and bioprinting.^158^ Furthermore, photocrosslinking has become a powerful tool in chemical biology for identifying and mapping stable or transient interactions between biomacromolecules. Smith and co-workers used mass spectra of peptide photocrosslinking to explore the initial oligomeric structure of amyloid fibrils [Fig. 10(c)].^159,160^ The comparison of the abilities of three probes, including phenyl trifluoromethyldiazirine, benzophenone, and phenylazide, revealed that phenyl trifluoromethyldiazirine gave more accurate mass spectra results of amyloid nanostructures due to the higher carbene intermediate reactivity.

### B. Affinity-based photocrosslinking of peptides to biomolecules

**FIG. 11.**
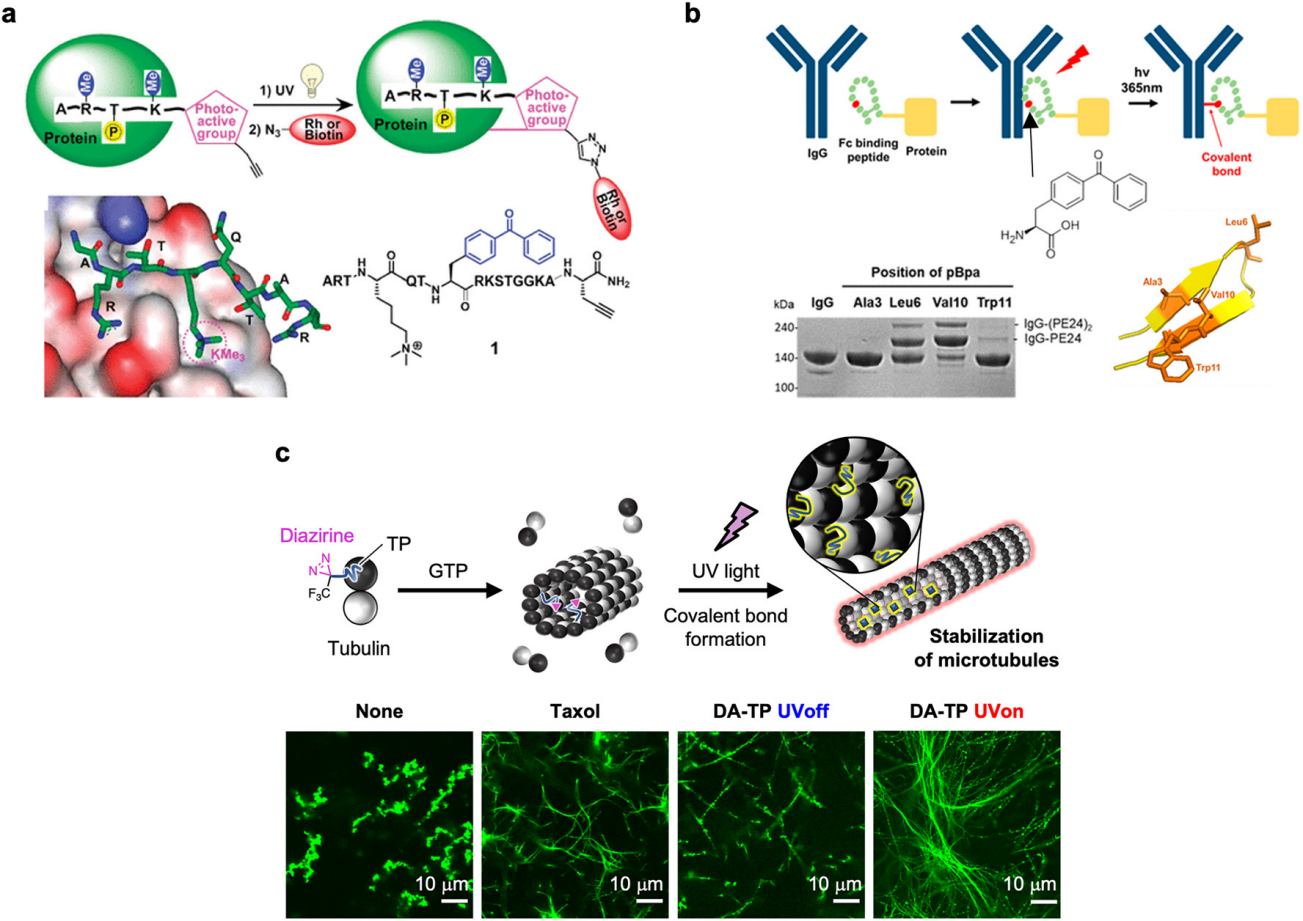
Affinity-based photocrosslinking of peptides to biomolecules. (a) Photo-affinity capture of proteins that recognize trimethylated lysine-4 of histone H3. Reproduced with permission from Li and Kapoor, J. Am. Chem. Soc. **132**, 2504–2505 (2010). Copyright 2010 American Chemical Society. (b) Site-specific IgG conjugation by peptide-directed photocrosslinking. Reproduced with permission from Park *et al.*, Bioconjugate Chem. **29**, 3240–3244 (2018). Copyright 2018 American Chemical Society. (c) Light-induced microtubule stabilization by the incorporation of diazirine-conjugated Tau-derived peptide (DA-TP) and subsequent UV light irradiation. CLSM images of GTP microtubules. Reproduced with permission from Watari *et al.*, Chem. Commun. **58**, 9190−9193 (2022). Copyright 2022 The Royal Society of Chemistry.

Photocrosslinking is a useful approach to covalently conjugate peptides to target biomolecules, and the reactivity was widely explored.^161,162^ In particular, a peptide probe that captured proteins that recognize histone post-translational modification was developed by conjugating a benzophenone moiety to the N-terminal fragment of trimethylated histone H3 at lysine-4 [Fig. 11(a)].^163^ The *in vitro* analysis revealed the covalent binding of the probe to a targeted protein, plant homeodomain (PHD) finger of ING2 by UV light irradiation followed by conjugation with rhodamine azide. Endogenous ING2 and other histone-binding partners in cell lysates, in addition to the targeted protein, were captured using the probe. Additionally, the photocrosslinking method was applied to a site-specific conjugation of peptides/proteins to antibodies.^164,165^ In the approach by Park *et al.*, a Fc-binding peptide with a benzophenone moiety was developed for the conjugation to targeted immunoglobulin G (IgG).^165^ Efficient photocrosslinking to Her2-targeting IgG (trastuzumab) and FcIII-fused engineered *Pseudomonasexotoxin* endotoxin A (PE24) was observed by optimizing the positions of benzophenone introduced to the Fc-binding peptide called FcIII [Fig. 11(b)]. The constructed trastuzumab-PE24 conjugate exhibited cytotoxicity to Her2-overexpressing cell lines, probably by protein synthesis inhibition. The photocrosslinking method would be useful to conjugate various cargos into antibodies for therapeutic and diagnostic applications.

### C. Stabilization of cytoskeleton by photocrosslinking

Photoisomerization is a useful approach for modulating microtubule structures, as summarized in Sec. II D. One of the issues of photoisomerization is the reversible property that may induce moderate structural change of microtubules. The photocrosslinking technology, as another approach, was used for the light-induced microtubule stabilization [Fig. 11(c)].^166^ Diazirine was used as a photocrosslinking agent that formed a carbene upon UV light (365 nm) irradiation to form a covalent bond between TP and microtubules. TP containing diazirine at the N-terminus (DA-TP) was synthesized and TMR was subsequently conjugated (DA-TP-TMR). The binding of DA-TP-TMR to microtubules and covalent bonding formation upon UV light irradiation was confirmed by confocal laser scanning microscopy (CLSM) and SDS-PAGE. DA-TP itself stabilized GTP-microtubule structures, such as taxol. Interestingly, UV light irradiation to DA-TP-encapsulated microtubules induced longer and more rigid microtubule formation [Fig. 11(c)]. The results indicate that forming the covalent bond between DA-TP and microtubules by UV light irradiation generated long, rigid, and stable microtubules. Additionally, the binding of DA-TP-TMR to intracellular microtubules of HepG2 cells was confirmed by CLSM. UV light irradiation to the DA-TP-TMR-incorporated cells and subsequent incubation caused abnormal cell shapes, nuclear defects, and cell death. Thus, the binding of DA-TP-TMR to intracellular microtubules and the UV light-induced microtubule stabilization caused cell death. The peptide-based microtubule stabilization would be a new approach for manipulating cell structures and functions.

## V. PHOTODIMERIZABLE PEPTIDE MATERIALS

**FIG. 12.**
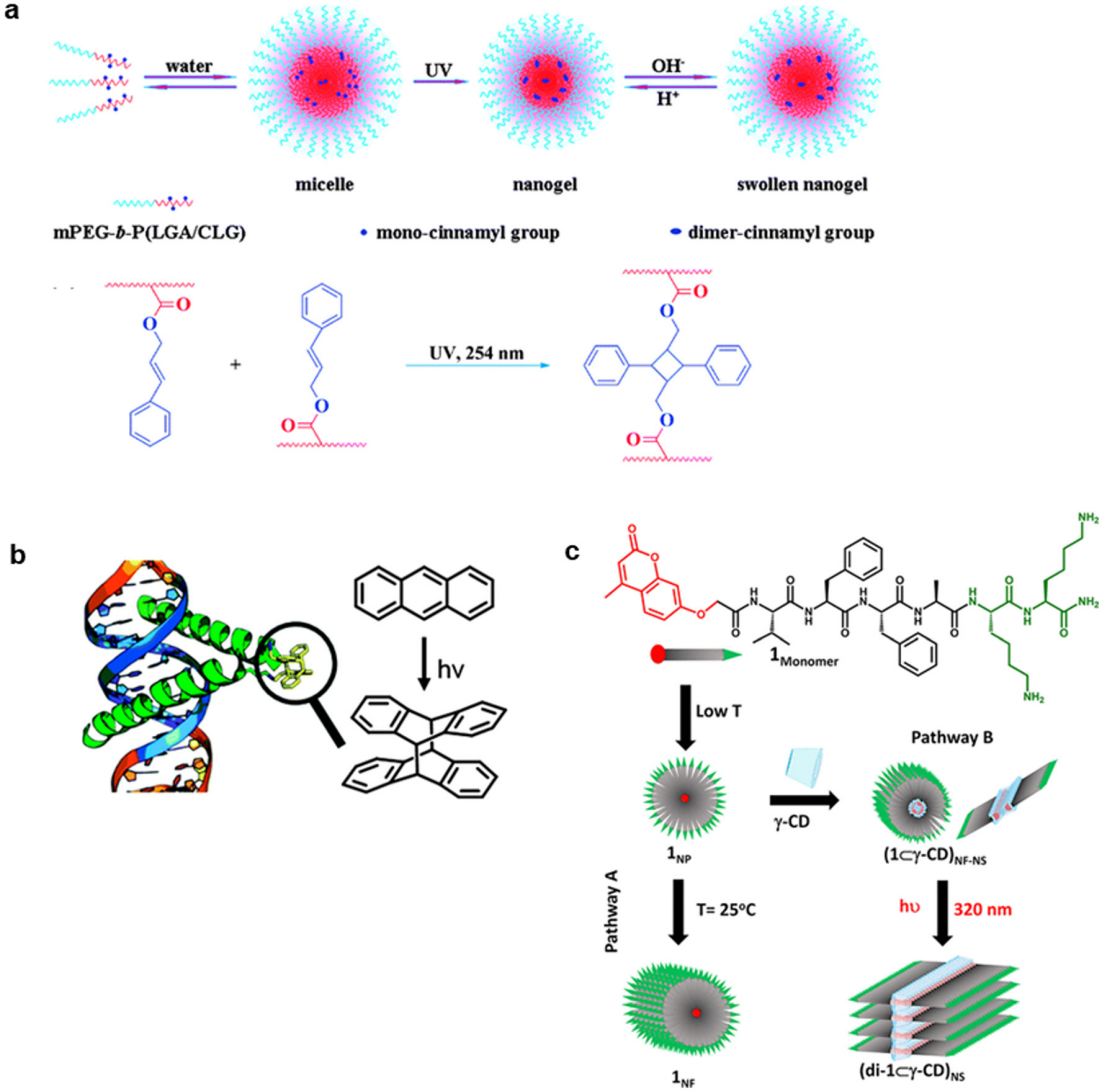
Photodimerizable peptide materials. (a) Photodimerized pH-responsive polypeptide nanogel. Reproduced with permission from Ding *et al.*, J. Mater. Chem. **21**, 11383–11391 (2011). Copyright 2011 The Royal Society of Chemistry. (b) Light-induced sequence-selective DNA binding of peptide by anthracene photodimerization. Reproduced with permission from Bullen *et al.*, Chem. Commun. **51**, 8130–8133 (2015). Copyright 2015 The Royal Society of Chemistry. (c) 2D peptide nanosheet formed by 4-methylcoumarin photodimerization in γ-cyclodextrin. Reproduced with permission from Joseph *et al.*, ACS Appl. Mater. Interfaces **11**, 28213–28220 (2019). Copyright 2019 American Chemical Society.

Photodimerization reactions can also be useful in controlling the biological function and self-assembly behavior of peptides although the examples are relatively less.^167–171^ Ding *et al.* developed pH-responding polypeptide nanogels that are stabilized by photodimerization between the cinnamyloxy groups [Fig. 12(a)].^168^ Diblock copolymer that comprise of poly(ethylene glycol monomethyl ether) (PEG) and poly(L-glutamic acid-co-γ-cinnamyl-L-glutamate) self-assembled into polymer micelles with PEG shell and γ-cinnamyl-L-glutamate core. The photodimerization through [2 + 2] cycloaddition between the cinnamyloxy groups afforded the cross-linked nanogels of approximately 30 nm in size. The release of the encapsulated antibiotic rifampicin in the cross-linked nanogel was controlled by nanogel swelling in response to pH. Bullen *et al.* showed that photodimerization of transcription factor GCN4 through [4 + 4] cycloaddition between the anthracenes induced sequence-specific and strong DNA-binding [Fig. 12(b)].^169^ The anthracene photodimerization only occurs after the conjugates are preorganized on the correct DNA sequence, thus it made the process very specific and controllable. Joseph *et al.* developed an Aβ42 amyloid nucleating core peptide modified with photodimerizable 4-methylcoumarin [Fig. 12(c)].^170^ The peptide conjugate self-assembled into 1D nanofibers via nanoparticle. In contrast, the photodimerization of two 4-methylcoumarin moieties that are included in γ-cyclodextrin resulted in morphological conversion to free-standing 2D nanosheets. The structural and temporal control to differentiate the pathway was used to tune the mechanical strength of hierarchical hydrogel materials.

## VI. SUMMARY AND OUTLOOK

This review outlined that the photocontrol of self-assembled materials and interactions with biomolecules by photoresponsive peptides has made significant progress over the past 30 years. Photoresponsive peptides were demonstrated to control the formation and dissociation of peptide assemblies, gene expression, protein-drug interactions, protein–protein interactions, deformation and motility of liposome, structure and stability of cytoskeletons, and cell functions. Many of the building units for incorporating photochromic, photocleavable, and photocrosslinking molecular units into peptides are available or can be readily synthesized. Photocontrol of biological systems through “optogenetics” using photoactive proteins has recently made remarkable progress,^172^ but photoresponsive peptides based on synthetic chemistry have the advantage of diversity in design and ease of preparation.

Developing photoresponsive peptides that respond faster and have higher spatiotemporal resolution is a future challenge. For the purpose, the optimization of structures of photoresponsive peptides is important. In addition to high-throughput screening methods, such as phage display, recent development of artificial intelligence-based technologies will help the generation of new photoresponsive peptides with superior properties such as high ON/OFF photoresponse, targetability, and self-assembly. The low stability of peptides due to proteolytic degradation may be problematic for the biological applications. Constraints of peptide conformations by cyclization and stapling and use of D-amino acids, β-peptides and peptoids are useful approaches to overcome the problem. They will help develop technologies for photo-controlling various biological systems, such as signal transduction, neuronal activation, molecular transport through channel proteins, cell division and differentiation, and internalization into cells. The use of photosensitive peptides to perform phototherapy may become possible for diseases, such as cancer, protein misfolding, and neurological. Photoresponsive peptides may also enable other *in vivo* applications such as tissue engineering, diagnostics, and immunomodulation. The challenge in using photoresponsive peptides *in vivo* is to develop molecules that respond to near-infrared light with high biopermeability. From the perspective of materials chemistry, creating smart molecular machines, molecular robots, and artificial cells is expected by incorporating photoresponsive peptides into multi-component molecular integration devices.^173^ For material applications of photoresponsive peptides, it is important to improve the stability and durability, understand structure-function relationships, and incorporate into other materials. Such integrated artificial systems that contain photoresponsive peptides will also be useful as next-generation smart DDS materials and can also help develop medicine.

## Data Availability

The data that support the findings of this study are available from the corresponding author upon reasonable request.
